# Novel Applications of Starch and Starch Derivatives in the Food and Alcoholic Beverages Industry: A Review

**DOI:** 10.3390/foods15020277

**Published:** 2026-01-12

**Authors:** Alice Vilela, Berta Gonçalves, Carla Gonçalves, Fernanda Cosme, Teresa Pinto

**Affiliations:** 1 Chemistry Research Centre-Vila Real (CQ-VR), Department of Agronomy, School of Agrarian and Veterinary Sciences, University of Trás-os-Montes and Alto Douro, Quinta de Prados, 5001-801 Vila Real, Portugal; 2 Centre for the Research and Technology of Agroenvironmental and Biological Sciences (CITAB), Institute for Innovation, Capacity Building and Sustainability of Agri-Food Production (Inov4Agro), Biology and Environment Department, School of Life Sciences and Environment, University of Trás-os-Montes and Alto Douro, Quinta de Prados, 5000-801 Vila Real, Portugal; bertag@utad.pt (B.G.); carlagoncalves@utad.pt (C.G.); tpinto@utad.pt (T.P.); 3 Unity RISE-HEALTH—Rede de Investigação em Saúde, University of Trás-os-Montes and Alto Douro, Quinta de Prados, 5000-801 Vila Real, Portugal; 4 Chemistry Research Centre-Vila Real (CQ-VR), Biology and Environment Department, School of Life Sciences and Environment, University of Trás-os-Montes and Alto Douro, Quinta de Prados, 5000-801 Vila Real, Portugal; fcosme@utad.pt

**Keywords:** modified starches, encapsulation, bioactives, 3D printing, clean label

## Abstract

Starch and its derivatives have undergone substantial advancement in the food and beverage industry, driven by growing demand for improved functionality and health-promoting attributes. Native starches are widely used as thickeners and stabilizers; however, their applications are limited by deficiencies such as poor freeze–thaw stability. To overcome these constraints, a range of physical, chemical, and enzymatic modification techniques has been developed, yielding starches with tailored and enhanced properties. Recent innovations include polyphenol-modified starches, which improve physicochemical characteristics and confer additional health benefits, such as reduced digestibility and increased antioxidant activity—features that are particularly valuable for functional foods targeting hyperglycemia. Enzymatic modifications further enhance starch quality and processing efficiency, while chemically modified forms, such as oxidized and acetylated starches, improve emulsification and water-binding capacities in various processed foods. Starch nanoparticles have also gained attention as encapsulating agents and carriers for bioactive compounds, broadening their technological applications. In parallel, the exploration of unconventional starch sources derived from fruit-processing by-products supports sustainability efforts while introducing novel functional attributes. Collectively, these developments are contributing to the creation of healthier, more stable food products that align with consumer expectations and regulatory standards. The following sections of this article examine emerging applications of starch and its derivatives, with particular emphasis on their health benefits and sustainable production pathways.

## 1. Introduction

Due to their versatile functional properties, starch and its derivatives are crucial in the food and beverage industries. These biopolymers are extensively modified to enhance their usability across a range of applications, from food stabilization to the production of alcoholic beverages. Starch can be modified through various methods, including chemical, enzymatic, and physical processes, to improve its functional properties. Common modifications include oxidation, esterification, hydroxyalkylation, dextrinization, and cross-linking, which enhance properties such as viscosity, stability, and resistance to retrogradation [1,2]. Transglycosylases also rearrange glycosidic linkages, creating starch derivatives with novel properties, such as thermoreversible gel formation and improved digestibility [3].

Modified starches are widely used in the food industry for their stabilizing and thickening properties. They are essential in overcoming the limitations of native starches, such as loss of viscosity and syneresis during cooking and storage [1,4]. Starch derivatives, such as resistant starch and dextrins, are valued for their nutritional benefits, which are similar to those of dietary fiber, and are increasingly used in functional foods [5,6].

In the alcoholic beverage industry, starch derivatives play a crucial role in fermentation processes. They provide a source of fermentable sugars, which are essential for alcohol production. Modifying starch can influence fermentation efficiency and the quality of the final product, though specific details on this application are less frequently highlighted in the literature.

Despite the extensive use of starch derivatives, challenges remain, such as obtaining legislative approval for novel modifications and addressing processing and storage issues [1,5]. Future research will likely focus on developing tailor-made starch derivatives with specific functional and nutritional properties to meet the growing demand for functional foods and beverages [1,5].

This review provides an overview of recent advances in the application of starch and its derivatives in the food and beverage industries, with a specific focus on fermented beverages. Starch and its derivatives are indispensable in the food and alcoholic beverage industries because they can be modified to enhance functionality. Ongoing research and development aim to overcome limitations and further expand their applications.

## 2. Starch and Starch Derivatives: Composition, Origin, and Applications

Starch, produced by plants during photosynthesis as an energy reserve, is obtained from various sources, including corn, potatoes, cassava, rice, wheat, peas, quinoa, sweet potatoes, dried beans, and ref. [7]. Starch is a natural polysaccharide made up of glucose units linked by glycosidic bonds, and is predominantly composed of amylopectin (poly-α-1,4-D-glycopyranoside and α-1,6-D-glycopyranoside, insoluble in water) and amylose (poly-α-1,4-D-glycopyranoside, soluble in water), in addition to various other short-branched polysaccharides with the ability to form gels [8,9,10].

Amylopectin is a branched polysaccharide, always with short chains, while amylose, apart from very long polysaccharide chains, rarely has branches [11]. Depending on the origin of the starch, the amylose and amylopectin contents differ; likewise, elongation and resistance increase with increasing amylose content. Higher amylose increases tensile strength (resistance). Because the amylose network is dense and well-ordered, it resists deformation more effectively. Materials with higher amylose content, therefore, exhibit greater tensile strength or “resistance” when mechanical force is applied—also, higher amylose increases elongation (ability to stretch). Although amylose strengthens the structure, it also provides flexibility. The linear chains can slide past one another to some degree before breaking, allowing the material to elongate more under tension. In contrast, highly branched amylopectin restricts chain mobility, leading to greater brittleness [12]. Amylose and amylopectin are not naturally found as isolated entities. Instead, they are organized into insoluble, aqueous-soluble complexes that form aggregates with semi-crystalline characteristics, known as starch granules. The structural organization of starch granules consists of an amorphous core, mainly composed of amylose, surrounded by layers with semi-crystalline properties. These layers consist of repetitive units containing amylopectin and amylose, arranged alternately in crystalline and non-crystalline zones, known as lamellae [13]. Depending on their origin, starch granules can vary significantly in shape, with a wide range of dimensions (1–100 μm), and exhibit different morphological configurations, including spherical, oval, rounded, shell-shaped, or irregular shapes [5,14]. In addition, their characteristics vary significantly depending on various agronomic factors, including plant variety, prevailing climatic conditions during cultivation, soil composition and fertility, among others. The concentration of starch can exceed 70% in cereal grains, while dry beans generally have between 36 and 47% [15,16].

**Figure 1:**
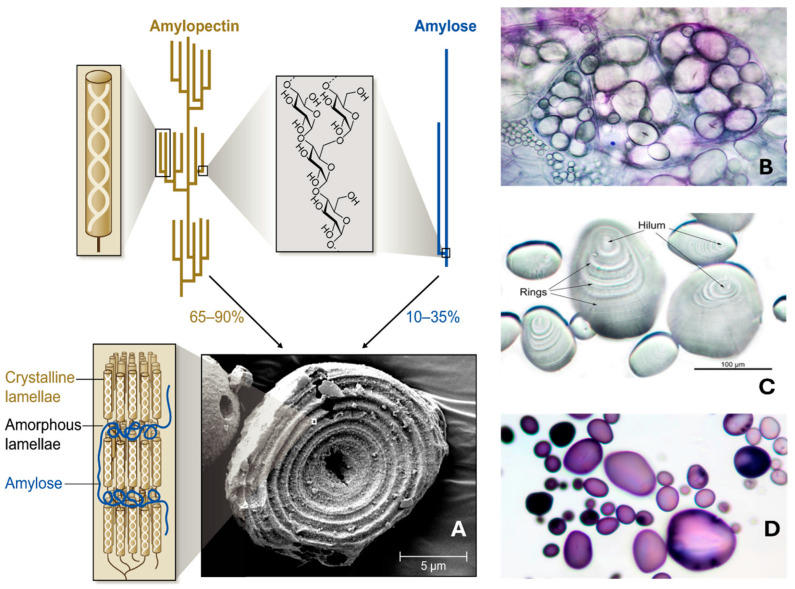
(**A**) Molecular architecture of starch granules; (**B**) amyloplasts of potatoes (brightfield microscopy, 100×); (**C**) starch grains’ central hilum; (**D**) potato starch grains stained with Lugol’s iodine (magnification of 200×). Composite image (**A**) was retrieved from Seung [17]; (**B**–**D**) were retrieved from Berdan [20]. Both references are open source, and images may be used with proper citation.

Figure 1A shows the molecular architecture of starch granules, revealing a complex arrangement of macromolecules [17]. Starch from grains, fruits, and other sources is enzymatically hydrolyzed into sugars, which are then fermented by yeasts, such as *Saccharomyces cerevisiae*, to produce ethanol. This process is fundamental in the production of beers, whiskies, and other spirits, with the efficiency and quality of fermentation depending on the type and treatment of starch used [18,19]. The use of enzyme preparations (e.g., amylase, amyloglucosidase) to break down starch into fermentable sugars has largely replaced traditional malt-based saccharification, improving process efficiency and reducing costs, though sometimes at the expense of flavor complexity [19].

In Figure 1B, it is possible to observe that within the amyloplasts of potatoes (specialized organelles that are responsible for the synthesis and storage of starch), a wide variety of starch grains, each exhibiting unique sizes and shapes, can be present. Starch grains are characterized by a central hilum (Figure 1C) around which concentric layers of protein are deposited. The presence of starch can be detected using Lugol’s iodine as a staining agent (Figure 1D) [20].

Modified starches can enhance the colloidal stability of alcoholic beverages by adsorbing and precipitating polyphenolic compounds, which helps prevent haze and sediment formation during storage, especially in fruit-based and fortified drinks [21]. Starch-based polysaccharides are utilized to produce dehydrated alcoholic beverage powders that can be reconstituted with water, thereby retaining flavors and aromas from various sources [22].

Beyond these industrial applications, starch derivatives are used to develop biosensors for rapid and accurate monitoring of glucose, ethanol, lactate, and starch content in alcoholic beverages, thereby aiding process control and quality assurance [23].

Regarding sensory characteristics, chemically modified starches (e.g., citrates, lactates) influence the texture, viscosity, and gel strength of beverages, thereby affecting mouthfeel and stability, especially in specialty or novel alcoholic products [24]. Moreover, the presence of starch and its derivatives in sugar used for alcoholic beverages can promote floc formation, impacting clarity and stability [25].

In the following sections of this article, we will highlight the innovative uses and applications of starch and its derivatives, including the health benefits of starches and the sustainable processes used to produce more nutritious, flavor-rich, and healthy foods and beverages.

## 3. Encapsulation of Bioactives & Nutraceuticals

The encapsulation of bioactives and nutraceuticals using starch and its derivatives is an emerging, promising application across the food and beverage industries. This technology leverages the structural, functional, and biodegradable properties of starch-based materials to protect, stabilize, and control the release of sensitive compounds, including vitamins, polyphenols, omega-3 fatty acids, probiotics, flavors, essential oils, antioxidants, and bitter-masking agents, as well as poorly water-soluble nutraceuticals such as lutein, lycopene, and curcumin [26].

Compared with lipid-based delivery systems, polysaccharide-based systems—such as those derived from starch—can be tailored to encapsulate a wide range of hydrophilic and hydrophobic compounds through appropriate modifications. Unlike lipid or protein carriers, starch-based systems are more thermally stable, making them better suited as encapsulating materials under high-temperature processing conditions, where lipids may melt, and proteins may denature. Starch offers several practical advantages: it is generally recognized as safe, cost-effective, non-allergenic, and neutral in flavor, and can be obtained from various plants such as corn, potato, tapioca, and rice. Comparative studies have shown that starch-based systems can achieve higher encapsulation efficiency and offer greater protection for sensitive ingredients—such as flaxseed oil and flavor compounds—when exposed to harsh environmental conditions, outperforming protein- and gum arabic-based alternatives in some cases [26,27].

Starch is a highly adaptable biopolymer that can be functionally modified to enhance its performance in food systems. Native starch has limited functionality. However, through chemical or physical modifications—such as esterification, etherification, or cross-linking—its properties can be significantly improved. These modifications enhance emulsification, gelation, and film-forming capabilities, making starch suitable for various encapsulation and delivery applications in the food and beverage industry [28,29].

Modified starches also offer excellent barrier and controlled release properties. They can protect sensitive bioactives and ingredients from environmental stressors such as heat, oxygen, light, and pH fluctuations, ensuring their stability and targeted release, for example, in the gastrointestinal tract or during cooking [30]. A range of starch-based materials is used in such applications, including native starch for basic encapsulation; octenyl succinic anhydride (OSA)-modified starches for efficient emulsification; starch nanoparticles and nanocrystals for enhanced surface area and stability; hydrogels and aerogels for prolonged release; and cyclodextrins—especially β-cyclodextrin—for encapsulating volatile flavors and aromas due to their unique cyclic structure [26,27].

**Figure 2:**
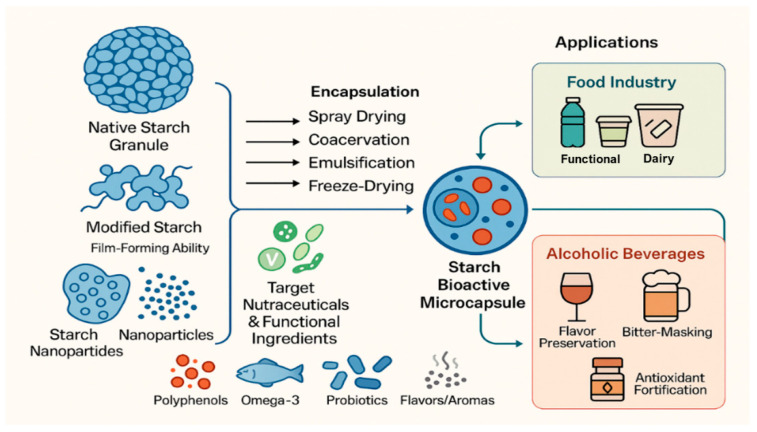
Schematic representation of starch and starch derivative applications in the food and alcoholic beverage industries by encapsulation of bioactives and nutraceuticals. Encapsulation techniques using starch and starch derivatives include spray-drying (most common for maltodextrins and OSA starch), spray-chilling/spray cooling, coacervation, extrusion, inclusion complexation (for cyclodextrins), nanoprecipitation (for starch nanoparticles), freeze-drying, and emulsion-based encapsulation [26,27,28,29,30,31,32,33,34,35,36,37,38,39]—figure made by the authors.

Starch-based encapsulation systems have diverse applications across the food and beverage industries due to their ability to protect and deliver sensitive bioactive compounds (Figure 2). In the food sector, they are widely used in functional foods to fortify omega-3 fatty acids [31], vitamins, and polyphenols [32], thereby ensuring their stability and bioavailability [33]. In bakery and dairy products, these systems help safeguard heat-sensitive bioactives during processing [34]. In beverages such as juices and sports drinks, starch-based carriers enhance clarity, improve flavor stability, and support effective delivery of nutrients [35]. Furthermore, starch’s natural origin makes it an ideal component in clean-label formulations, appealing to health-conscious consumers. In alcoholic beverages, encapsulation techniques are employed to mask bitterness in fortified wines, beers, or spirits, enhance the stability of volatile flavor and aroma compounds during storage, and enable the controlled release of flavors or nutraceuticals in ready-to-drink products and mixers [36,37].

Despite the growing potential of starch-based encapsulation systems, several challenges remain that must be addressed to enhance their commercial viability. One key issue is encapsulation efficiency, which tends to be lower when using native starch but can be significantly improved with chemically or physically modified variants. Another critical aspect is the ability to precisely control release kinetics across varying pH and temperature conditions, which is essential for targeted delivery in different food matrices or during various stages of digestion. Additionally, regulatory acceptance is vital, particularly for modified starches that must meet safety and labeling standards, such as those outlined by the EU’s E-number system. Finally, for large-scale adoption, encapsulation techniques such as spray-drying, freeze-drying, and coacervation must be optimized to ensure both scalability and cost-effectiveness without compromising the integrity or functionality of the encapsulated compounds [30,38,39].

## 4. Fat Replacers in Low-Calorie Foods

An unhealthy diet with foods high in saturated and trans fats, salt, and sugar (especially in sweetened drinks) was recognized as a primary risk factor for non-communicable diseases such as obesity, diabetes, and cardiovascular diseases [40]. The food industry initially developed low-fat products to meet the specific dietary needs of consumers with diabetes or other health conditions. However, the demand for these products has increased significantly among broader target audiences, including individuals concerned with preventing disease, losing or maintaining weight, or adopting a healthy lifestyle. Thus, the food industry was encouraged to invest more in developing low-fat products with pleasant sensory characteristics at competitive prices, as mandated by current legislation [41]. The use of starch-based fat replacers in low-calorie foods is a well-established yet continuously evolving application, due to their ability to form gels that simulate the smooth texture of fat, their versatility, low cost, widespread acceptance as a food ingredient, and compatibility with clean-label and plant-based product trends. These fat replacers primarily mimic the mouthfeel, texture, and functionality of fats while significantly reducing their caloric content [39].

Starch-based fat replacers can be produced either chemically, as modified starch, or enzymatically, as maltodextrins. The more commonly used types are listed in Table 1.

These fat replacers generally imitate the sensory and the physical–chemical characteristics of fat globules. They can also replicate fat by forming gel-like structures that hold added water and gradually release it, as fat does during consumption [42]. In this context, replicate fat refers to the ability of starch-based systems to mimic critical functional roles of fats in foods—such as providing creaminess, lubrication, viscosity, and contributing to flavor release. When hydrated, starch-based gels create a smooth, cohesive texture and a controlled water-release profile that resembles the melting and breakdown of fat globules during mastication, thereby producing a similar mouthfeel. Native starch typically exhibits limited functionality in food applications due to factors such as pH sensitivity, structural fragility, high retrogradation tendency, and poor thermal stability during processing. As a result, the food industry often opts for modified starches, tailored to meet specific performance needs through chemical, physical, or enzymatic modifications [42].

In recent decades, starch has been altered using different techniques. These modifications generally fall into four main categories: chemical, physical, enzymatic, and genetic [28]. Promising emerging technologies such as ultrasound, high-pressure processing, high-homogenization processing, pulsed electric field, and cold plasma have been studied [28,29].

Some examples of applications in the food industry include dairy alternatives, such as low-fat butter spread/margarine [43], low-fat milk-type products, low-fat yogurt [44,45], low-fat cheese [46], low-fat mayonnaise [47,48], and low-calorie ice cream/desserts [49]. Other food applications include the use in dressings and sauces, stabilizing emulsions, and replacing oil in low-calorie versions of food products [50]. Additionally, it is used in meat products to improve juiciness in lean formulations or meat substitutes [51,52]. Potential uses in the alcoholic beverage industry, with niche applications, include low-calorie cocktail bases (e.g., creamy or thick mixers with reduced fat using starch-based systems) and mouthfeel enhancement in non-alcoholic or light alcoholic beverages where fat would traditionally contribute to the sensory profile (e.g., creamy liqueurs).

Starch-based fat replacers offer several advantages that make them attractive for use in low-calorie and reduced-fat food products. Specific forms, like resistant starch, can also enhance dietary fibre content and promote satiety. Additionally, these starch derivatives used as additives can improve product stability over shelf life by maintaining texture and moisture, contributing to better quality retention [38]. Their plant-based origin and functional versatility make them suitable for clean-label formulations, aligning with sustainability and health-driven market trends [39,42].

Despite these benefits, starch-based fat replacers face specific challenges that may limit their use in some applications. Native starches can impart undesirable flavors or aftertastes and often lack the thermal and pH stability required in many food processing environments. While these limitations can be addressed through chemical or physical modification, starches may still fall short in mimicking the full flavor release and lubrication properties provided by fats. As such, further innovation is needed to enhance their sensory performance and broaden their applicability in complex food systems.

## 5. Edible Coatings and Films

The accumulation of plastic waste, which takes hundreds of years to decompose, has prompted the search for sustainable, environmentally friendly alternatives. Currently, there is growing global concern about the excessive use of petrochemical-based polymers [5]. In this context, the demand for biodegradable films and coatings derived from bio-based, environmentally friendly materials has driven research and development of starch-based films [53]. These materials possess promising characteristics, including biodegradability, non-toxicity, and sustainability, which align with the principles of the circular economy, a crucial consideration in the context of increasing pollution and environmental degradation [53]. In addition, they are semipermeable to lipids, carbon dioxide, and oxygen, and are odorless, colorless, and tasteless [54]. The European Commission has set targets for the complete recycling or reuse of plastic packaging by 2030 [55]. Currently, 53% of packaging, especially that used in the food industry, is biodegradable [56].

Several physical and chemical modification strategies have been applied to optimize the functional properties of starch-based films and coatings [57,58]. At the same time, the incorporation of natural and functional additives—such as spices, herbs, vegetables, seeds, and fruits—has been explored as a complementary approach to developing active and smart packaging [59]. Among the frequently used additives, nanoparticles and essential oils stand out, whose introduction aims to improve the barrier, mechanical, and bioactive properties of starch materials [60,61,62].

According to Ibáñez-García [63], the properties of biodegradable and thermoplastic polymers stem from the two main polysaccharides that comprise starch. As a widely available, renewable biopolymer, starch plays a significant role in the materials industry, particularly as thermoplastic starch (TPS). TPS is highly sensitive to moisture, and its thermal properties are influenced by water content. Due to its biodegradability, low cost, and natural origin, TPS serves as an alternative to synthetic polymers.

However, Bangar et al. [64] note that the direct use of starch in the food industry is limited by its inherent weaknesses in its natural form. These limitations include insolubility in cold water, high moisture sensitivity, low structural resistance, and susceptibility to degradation. Additionally, the high content of hydroxyl groups in starch contributes to its hydrophilicity, reducing its water resistance and mechanical performance under high-humidity conditions. Therefore, enhancing the mechanical strength and barrier properties of starch-based packaging materials is crucial. To address these shortcomings, strategies such as the chemical modification of starch or its combination with other compounds have been widely employed [65,66].

A study by Tafa et al. [7] aimed to optimize the mechanical properties of starch-based films by incorporating plasticizers and agar into the starch polymer matrix. They used 5 g of tef starch, 0.4 g of agar, and 0.3% glycerol in their formulations. This approach resulted in a significant improvement in the films’ mechanical properties, making them more suitable for applications requiring both strength and flexibility. The incorporation of these compounds enhanced interactions among the material’s constituents, helping to overcome the intrinsic limitations of using starch alone.

Among various natural polymers, starch is used for food packaging due to its low cost, abundant availability, edibility, and degradability [67]. In addition to enhancing food quality, starch-based biodegradable films have attracted increasing interest in the scientific community, particularly for their direct interaction with the food they coat. These coatings are recognized for their significant effectiveness as barriers against oxygen, water vapor, and ultraviolet radiation [65]. Furthermore, when they incorporate bioactive compounds, such as polyphenols, essential oils, or nanoparticles, these materials can exhibit antioxidant and antimicrobial properties, making them particularly advantageous for preserving highly perishable food products. Antioxidant, antimicrobial, barrier, and mechanical resistance characteristics are key parameters for the efficient application of starch-based biodegradable materials in the food packaging sector, significantly contributing to the improvement of quality, safety, and shelf life of packaged foods [68]. Variability in the physicochemical properties of foods, such as biological origin, moisture content, and texture, results in different conservation requirements.

Therefore, the development of starch films with functionally adjusted characteristics becomes essential, not only to optimize their technological performance but also to ensure better adaptation to the specific preservation needs of different food product categories [69]. In recent decades, scientific research on starch-based polymeric films has primarily focused on characterizing their sources, the processing methods used in their formulation, and the potential opportunities and limitations of using starch materials [70]. Take, for example, the case of blueberry coating to prevent water loss during storage. In this specific situation, curcumin nanoemulsions were added to starch-based films, thereby providing a better barrier to water loss and an environment in which high carbon dioxide concentrations inhibit blueberry respiration [3]. Additionally, a study by Fakhouri et al. [71] developed an edible biofilm composed of starch and gelatin to prolong the post-harvest storage of Crimson red grapes under refrigeration. The application of this coating proved effective in maintaining the fruit’s attractive visual appearance for up to 21 days while also reinforcing its structural integrity. Furthermore, sensory tests with consumers indicated that the coating did not compromise the organoleptic characteristics of the grapes, demonstrating good acceptance of flavor and texture. Another example is the application of biodegradable packaging formulated from starch films, incorporating cellulose extracted from corncobs and cassia seed oil, which has shown promising results in preserving green grapes. According to experimental data, this formulation led to a significant reduction in degradation, slowing down the deterioration process and helping to maintain the freshness of the grapes during storage. The study, therefore, highlights the dual positive impact of the adopted approach: improving the functionality of starch-based biodegradable packaging and promoting the circular economy through the reuse of corncob material [39].

Table 2 summarizes recent studies highlighting the beneficial effects of starch-based edible coatings on the shelf life of various foods.

Starch derivatives, obtained via chemical, physical, or enzymatic modifications such as esterification, oxidation, or cross-linking, are widely used in edible coatings and films to overcome limitations of native starch, including brittleness and high hydrophilicity [2,94]. These modifications improve film-forming ability, flexibility, and mechanical stability, while reducing retrogradation and enhancing barrier properties [2]. Derivatives such as octenyl succinic anhydride, starch, and starch acetates increase hydrophobicity and facilitate the incorporation of bioactive compounds, thereby supporting multifunctional coatings that extend shelf life and improve food quality [95,96,97].

In starch-based coatings, the relative proportion of amylose to amylopectin is crucial to the material’s functionality, as it directly affects the microstructure of the resulting films. Starches with a high amylose content, whose linear conformation favors the formation of helical structures, tend to produce matrices with more densely organized domains and a more crystalline nature, giving them superior mechanical and barrier properties [95,96,97]. In this context, the results presented in [79] demonstrate that coatings produced with high-amylose starch were more effective at preserving firmness and reducing mass loss in strawberries during storage than formulations based on medium-amylose starch. Additionally, it was observed that plasticizing the films with sorbitol resulted in superior resistance to water vapor permeability compared with glycerol [71,79]. In addition, Sagnelli et al. [97] report the application of starch consisting exclusively of amylose, produced through genetic engineering, to produce starch materials with significantly improved functional properties, highlighting the biotechnological potential for modulating the characteristics of starch films. Thus, improving starch-based biodegradable films is not only a response to environmental challenges but also an opportunity to innovate materials that promote sustainability [67].

Despite advances in physical, chemical, and biotechnological modification techniques, starch-based films and packaging still face considerable challenges in their mechanical and barrier properties, which remain inferior to those required by conventional plastic packaging. There is therefore a need for further scientific and technological studies to optimize these characteristics, favoring green technologies and processes with low environmental impact to minimize additional waste and reduce the ecological footprint of production. Finally, quantifying the carbon footprint of the life cycle of starch-based packaging is crucial for validating the sustainability often attributed to these materials [54].

In addition to their use in the food industry, starch films are also being utilized in the beverage industry as sustainable, biodegradable alternatives to traditional packaging. Their applications range from intelligent spoilage detection to edible, water-soluble packaging for instant beverages. Starch films offer eco-friendly, functional, and innovative solutions for beverage packaging and quality monitoring (Table 3).

Recently, there has been growing interest in using deep eutectic solvents (DES) and their natural variants (NADES) as eco-friendly alternatives to conventional plasticizers when processing biopolymers, such as thermoplastic starch [106,107,108]. NADES are eutectic mixtures formed by hydrogen bond donors and acceptors—generally compounds of natural origin—which are promoted in food and sustainable packaging applications due to their low toxicity, high biodegradability, and “green solvent” properties [109]. Studies demonstrate that incorporating DES into starch matrices can significantly enhance the mechanical and physical properties of resulting thermoplastic films compared to traditional plasticizers. For instance, choline chloride-based NADES, combined with oxalate or ascorbic acid, demonstrates superior plasticizing efficiency compared to glycerol in cassava starch films, reducing crystallization and promoting greater elasticity and ductility at lower plasticizer concentrations [110].

Although the primary focus of Alsaidi and Thiemann [106] is the application of NADES in food extraction, preservation, and packaging, the authors also highlight the role of these environmentally friendly solvents as functional components in polymeric packaging materials. This includes integrating them into biopolymer matrices to modify the materials’ physicochemical and barrier properties. Other studies, such as those by Skowrońska & Wilpiszewska [108], demonstrate that DES/NADES can act as plasticizers for starch, disrupting intermolecular polymer bonds and reducing crystallinity. This improves the flexibility and processability of starch-based films and materials compared to conventional plasticizers such as glycerol. This innovative use of NADES aligns with the principles of sustainable chemistry. It offers a promising approach to developing starch films and coatings with enhanced mechanical and barrier properties, thereby expanding the potential applications of biopolymers in the food and packaging industries [109].

## 6. 3D Food Printing

The application of starch and starch derivatives in 3D food printing represents a cutting-edge trend in the food industry, driven by the need for personalization, sustainability, and innovation in food design [111,112]. 3D food printing with starch-based inks enables the production of customized foods, both in terms of shape and nutritional profile, which is particularly valuable for populations with special dietary requirements, such as the elderly, children, or patients with dysphagia [113,114]. The technology also opens up possibilities for novel textures and multi-layered products that are difficult or impossible to achieve with traditional food processing methods [115].

**Figure 3:**
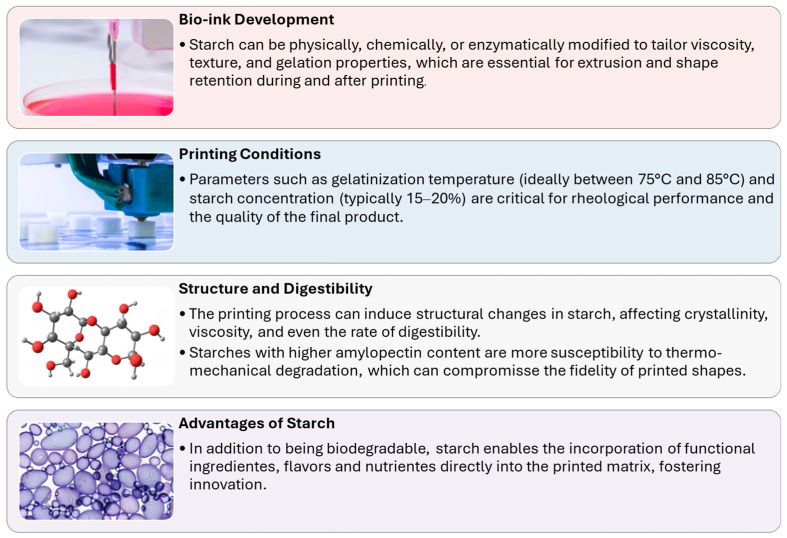
Exploring starch as a key component in 3D food printing. Figure made by the authors.

Starch is particularly attractive as a base for edible bio-inks due to its natural abundance, affordability, and the ease with which its physicochemical properties can be tailored to specific applications [70,100,111,112,116]. Its ability to form gels, pastes, and films makes it highly suitable for extrusion-based 3D printing technologies, where the rheological behavior of the printing material is critical for shape fidelity and structural stability [117,118,119,120]. Recent studies have demonstrated that both native and modified starches can be engineered to achieve the desired viscosity, gelation, and mechanical properties necessary for successful 3D food printing, enabling the creation of complex, customized food structures while maintaining nutritional and sensory quality [121,122]. Starch can be physically, chemically, or enzymatically modified to achieve the desired viscosity, gel strength, and stability required for 3D printing processes (Figure 3). For example, pre-gelatinized or cross-linked starches are often used to enhance printability and maintain the intended shape after deposition [123,124]. Moreover, the gelatinization temperature, amylose-to-amylopectin ratio, and water content of starch-based formulations profoundly influence both the printability and final texture of 3D-printed foods [125]. These modifications not only enhance the mechanical properties of the printed structures but also enable the incorporation of additional nutrients, flavors, or functional ingredients, allowing for the creation of foods tailored to specific dietary needs or consumer preferences [121]. Artificial intelligence models, such as neural networks, are increasingly being applied to predict and optimize the performance of starch gels in 3D printing, thereby improving process efficiency and product quality [126].

Ji et al. [121] highlighted that the molecular structure of starch controls 3D printing performance and texture, with maize and wheat showing the best results. They emphasized the key role of specific chain lengths in extrusion, product shape, and resistance. Similarly, Mu et al. [127] demonstrated that increasing the oil content in β-carotene–loaded starch emulsion gels simultaneously improved extrudability and self-support during 3D printing by acting as an active filler and lubricant, while also enhancing microstructure and retention.

Modified starches, such as cross-linked or acetylated variants, have also been optimized to balance printability with sensory attributes. Indeed, Riar et al. [128] showed that starches from broken rice grains (PUSA-44, PR-106, and PR-114) were modified by hydroxypropylation and esterification. The degree of substitution was low (0.02–0.12), and modifications affected their properties. Acetylation and dual modification increased paste clarity, solubility, swelling power, and gel strength but reduced gel elasticity [128]. Cross-linking, in contrast, lowered solubility and swelling while improving paste clarity and gel strength. Moreover, octenyl succinic anhydride (OSA)-modified starches have been used as food additives for decades, with growing interest as new methods and applications emerge. Their unique structure provides valuable stabilizing, encapsulating, interfacial, thermal, nutritional, and rheological properties [129]. Pająk et al. [62] demonstrated that octenyl succinylated (OS) potato starch films incorporated with ethanolic extracts of honey bee products (HBE) exhibited enhanced thermal stability, increased hydrophobicity, and superior antioxidant properties compared to native starch films. Notably, propolis-enriched OS starch films showed the highest phenolic content and the most potent antimicrobial activity, highlighting their promising potential for food packaging applications, albeit with some compromises in optical and sensory characteristics. According to Gao et al. [130], corn starch (CS), octenyl succinic anhydride-modified corn starch (OSCS), and shell microgels prepared via water-in-oil inverse microemulsions demonstrated enhanced encapsulation efficiency, controlled release, and strong interactions with epigallocatechin gallate (EGCG), with OSCS showing the smallest particle size and promising potential as polyphenol encapsulating agents. Moreover, Sweedman et al. [129] showed that octenyl succinic anhydride-modified turmeric starches (O-MTSs) exhibited improved solubility, swelling, transparency, and emulsifying capacity for stabilizing Pickering emulsions compared to native turmeric starch, with structural changes confirmed by SEM, XRD, FT-IR, and thermal analyses. These advancements underscore starch’s adaptability in meeting both technical and consumer-centric requirements for applications ranging from personalized nutrition to dysphagia-friendly medical foods.

## 7. Clean-Label Thickeners

The increasing consumer demand for natural, transparent, and minimally processed foods has driven rapid expansion in the development and application of clean-label starch-based thickeners. Today’s shoppers are prioritizing simple ingredient lists, recognizable components, and avoiding additives perceived as artificial or chemically modified [131]. This shift in consumer preferences is reshaping the food industry, as manufacturers seek to build trust and offer products that align with the values of health-conscious, ingredient-aware consumers.

Clean-label foods, characterized by short ingredient lists and supported by innovative processing technologies, play a crucial role in reducing consumer skepticism and increasing willingness to pay a premium for products perceived as healthier or more natural [132]. In response, the food industry has increasingly turned to starch-based thickeners produced through physical or enzymatic methods, rather than chemical modification [133]. This strategic choice enables these ingredients to be declared as “starch” on product labels, thereby avoiding the need for E-numbers or additive classifications and fully meeting clean-label requirements [134,135].

Clean-label starch thickeners are typically manufactured using non-chemical processes such as pre-gelatinization, heat–moisture treatment, high-pressure processing, or enzymatic hydrolysis [136]. These advanced techniques modify the granular structure and functional properties of starch, enhancing its solubility, swelling capacity, and viscosity while preserving its natural state [137,138]. For instance, pre-gelatinized starches are produced by cooking and then drying the starch, resulting in an ingredient that can instantly thicken cold liquids, making them especially suitable for ready-to-eat foods, beverages, and convenience products [139].

Beyond traditional food applications, the versatility of clean-label starches is fueling innovation in emerging areas such as 3D food printing, where their unique rheological properties—including viscosity and structural stability—are leveraged to create customized, structurally sound food products. This convergence of clean-label trends with advanced food technologies is enabling manufacturers to deliver not only more natural and transparent products but also novel, personalized food experiences that meet evolving consumer expectations.

Clean-label starches have become indispensable ingredients in a diverse array of food and beverage products, including soups, sauces, dairy alternatives, fruit preparations, and bakery fillings (Table 4). In these applications, they are valued for their ability to impart desirable texture, enhance stability, and deliver a pleasant mouthfeel, all of which are critical for maintaining product quality and consumer satisfaction [140]. Unlike their chemically modified counterparts, clean-label starches are preferred for their consumer-friendly image, as they align with growing demands for natural, minimally processed ingredients and help manufacturers comply with increasingly stringent regulatory and marketing standards [141].

A key advantage of clean-label starches is their robust process stability, which includes resistance to heat, pH fluctuations, and mechanical shear during production and storage. These starches also exhibit reduced retrogradation—the tendency for starch gels to harden over time—and minimal syneresis—the release of water from gels—ensuring that products maintain their consistency, appearance, and sensory qualities throughout their shelf life [146].

The adoption of clean-label starch thickeners not only strengthens consumer trust and boosts product appeal but also provides manufacturers with a unique opportunity to stand out in a crowded marketplace [149]. By leveraging these ingredients, companies can cater to the preferences of health-conscious, ingredient-aware consumers who are willing to pay a premium for products perceived as more natural and transparent [150].

## 8. Fermentation Substrates

Starch and its derivatives are widely used as substrates for fermentation in various biotechnological processes, including the production of bioethanol, hydrogen, and value-added biochemicals. The choice of starch type, its structural modifications, and the fermentation method significantly influence the efficiency and outcomes of these processes.

Substrates include native starches from maize, pea, canna, potato, cassava, and lentil, as well as modified forms such as retrograded, debranched, hydroxypropylated, and cross-linked starches. These modifications alter digestibility and fermentation profiles [151,152,153,154,155,156].

Starch hydrolysis (using acids or enzymes) is a prerequisite for ethanol fermentation by yeasts. Substrates include soluble and insoluble starches, sorghum, sweet potato, rice, and potato waste. Recombinant yeasts with enhanced amylase activity improve the conversion of raw starch and ethanol yields [157,158,159].

Starch-rich agricultural residues (e.g., potato, cassava) serve as substrates for dark and photo-fermentation, with process optimization (e.g., substrate concentration, oscillation) significantly affecting hydrogen yield and production rates. Combining dark and photo-fermentation increases overall hydrogen yield and energy conversion efficiency [155,156]. Solid-state fermentation using starchy residues (e.g., cassava bagasse) supports the production of amylolytic enzymes by Bacillus species, with process parameters (moisture, pH, temperature) influencing yields [159].

The starch structure plays a crucial role in determining the efficiency and outcomes of alcoholic beverage fermentation. Changes in starch molecular structure directly affect sugar availability, fermentation rates, alcohol yield, and the sensory qualities of the final product.

Starch with higher short- and intermediate-chain fractions (e.g., from extrusion or certain adjuncts) is more readily hydrolyzed, resulting in higher levels of fermentable sugars (e.g., maltose and glucose) in the wort, which enhance fermentation rates and alcohol production [160,161]. In contrast, a higher proportion of long amylopectin chains or higher amylose content is negatively correlated with maltotriose release and ethanol yield. In contrast, shorter chains are more favorable for efficient fermentation [159,160].

Waxy starches, rich in amylopectin, ferment faster initially but slow down due to acid accumulation. In contrast, regular starches provide steadier fermentation. According to Yang et al. [162], sorghum starch is the main carbohydrate for baijiu production, where it is converted into ethanol and aromatics. Early in fermentation, waxy sorghum exhibits higher fermentation rates and saccharification power. However, these rates drop below those of regular sorghum in the later stages due to acid buildup, which inhibits enzyme activity and halts fermentation.

Regarding ethanol content and flavor compounds, altered sugar profiles (e.g., higher glucose or maltose) resulting from different starch structures can alter higher alcohols and esters, which in turn affect the aroma and taste of the beverage [159,163]. For example, extruded cassava starch increases the production of 2-phenylethyl alcohol, imparting a rose aroma to beer and enhancing sensory quality [159]. Also, the type and rate of sugar release from starch hydrolysis influence the concentration of fusel alcohols and other volatiles [160,163].

During fermentation, starch undergoes structural changes. Starch granules swell, rupture, and lose crystallinity as fermentation progresses, thereby affecting their viscosity and gelatinization properties and further influencing fermentation dynamics and product texture [119,164].

Additives, such as hydrocolloids, can improve the stability and texture of starch during fermentation [164]. Jin et al. [164] examined the effects of 1% xanthan gum (XG) and hydroxypropyl methylcellulose (HPMC) on the physicochemical properties and structure of triticale starch during fermentation. Results from frequency scanning and a rapid viscosity analyzer indicated that the addition of XG or HPMC during fermentation reduced the loss factor (tan θ) and increased peak viscosity. This suggests that the gel network’s strength was enhanced. Furthermore, microstructural and thermal analyses revealed that encapsulating triticale starch with XG or HPMC improved its thermal stability during fermentation.

Fermentation and modification of starches (e.g., using “sub-high” amylose maize starch) can increase resistant starch content and alter digestibility, with implications for health and industrial applications [151,152,153,154].

Using low-cost, abundant starchy substrates (including agricultural waste) supports sustainable production of biofuels and biochemicals. Modified starches can be tailored for specific fermentation outcomes, such as increased RS or targeted SCFA profiles [151,152,155,156,157,158]. However, some native starches require extensive hydrolysis or modification to enable efficient fermentation. The choice of substrate and process conditions is critical for maximizing yields and product quality [151,155,156,157,158,159].

Starch derivatives play a central role in the brewing process, influencing both fermentation efficiency and the sensory and nutritional qualities of beer. Recent research investigates the impact of various starch sources, their molecular structures, and processing methods on brewing outcomes.

Common starch adjuncts used in beer fermentation include cereals (such as barley, wheat, maize, rice, sorghum, oats, rye, and millet), pseudo-cereals (buckwheat, quinoa, and amaranth), and tubers (such as sweet potato and cassava). These can be added in various forms, such as whole grains, grits, malted, extruded, torrefied, or as syrups [160,165]. Starch-rich microalgae and high-amylose rice cultivars are being explored as novel adjuncts, offering unique sensory and nutritional profiles [166,167]. *Tetraselmis chui* microalgae were successfully integrated into the brewing process at a small scale as an active ingredient, producing microalgae-enriched beer containing up to 20% algal biomass. The addition of *T. chui* had a noticeable effect on the beer sensory properties. The 20% beer had the most intense profile, with green hues, a stronger seaweed flavor and aroma, marked syrupy notes, and a strong umami taste. According to the authors Carnovale et al. [166], these preliminary remarks on the profile of microalgae-enriched beer must be integrated in future studies, with a thorough sensory evaluation performed by a trained tasting panel.

In rice beer, the regular amylose-containing cultivars Samgwang and Hangaru and the high-amylose-containing cultivar Dodamssal were used as adjuncts by Park et al. [167]. Dodamssal rice beer had the least bitterness and the lowest levels of volatile components, such as acetaldehyde and ethyl acetate, which helped to diminish the oxidized and gluey smell. The characteristics of rice beers varied with the molecular structure of the ingredients, irrespective of amylose content.

Regarding brewing performance and beer quality, the molecular structure of starch derivatives affects the breakdown into fermentable sugars during mashing. Extrusion and other modifications can increase maltose and glucose yields, thereby enhancing fermentation efficiency and alcohol content [160,168].

The choice and processing of starch adjuncts influence aroma, flavor, and mouthfeel. For example, extruded grains and cassava can boost aroma compounds and esters, while specific rice cultivars can produce beers with rich flavors and lower bitterness [160,165,167]. Non-conventional starch sources, such as black rice or sweet potato, can increase polyphenol content and other nutritional characteristics in beer. Sweet potato in the form of purple sweet potato flakes increased the pink color, β-carotene content, and antioxidant activity in an Ale beer [169]. Some adjuncts also enable the production of gluten-free beer [165].

Besides the sensory profile imprinted by the starch source, advanced analytical techniques can distinguish beers by their starch source, aiding quality control and authenticity verification. Pieczonka et al. [170] investigated the impact of various starch sources on the beer metabolic signature using a non-targeted analytical approach. They analyzed a wide range of commercial beers, brewed with barley, wheat, corn, and rice, using direct infusion Fourier transform ion cyclotron mass spectrometry (DI-FTICR MS) and UPLC-ToF-MS. DI-FTICR-MS revealed both polar and non-polar metabolites linked to the starch sources, while UPLC-ToF-MS provided insights into molecular structures and isomeric separation. The analyses revealed clear differentiation among the beer samples by starch source. They identified the aspartic acid-conjugate of N-β-D-glucopyranosyl-indole-3-acetic acid as a potential marker for rice in brewing, which is helpful for quality control and food inspection.

## 9. Flavor Stabilization and Controlled Release of Flavors in Beverages

Starch and its derivatives are increasingly used in the beverage industry for flavor stabilization and controlled flavor release. These materials can encapsulate flavors, protect them from degradation, and modulate their release, thereby enhancing product quality and the consumer experience. Native and modified starches, including starch, cyclodextrins, maltodextrin, octenyl succinic anhydride (OSA) starches, and porous starch, are commonly used for flavor encapsulation due to their stability, cost-effectiveness, and versatility [26,171,172]. Combining starch with other compounds, such as tannic acid, can further enhance flavor retention and control release [173]. A good example is the modified food starch, commercially known as CAPSUL^®^, used as a wall material for encapsulating essential oils, which provides high encapsulation efficiency and flavor retention in beverages [174].

The mechanisms of flavor stabilization and controlled release that usually occur are (i) physical entrapment, where starch films and microcapsules physically trap flavor molecules, thereby protecting them from oxidation and volatilization [171,174,175]; (ii) hydrophobic interactions, where modified starches, especially OSA–starch, interact hydrophobically with volatile compounds, slowing their release [171]; (iii) microstructure effects, the structure of starch–protein or starch–sucrose matrices influences aroma distribution and release, with phase separation and water activity playing key roles [176]; and thermodynamic and kinetic factors: The release of flavors is influenced by the matrix composition, temperature, pH, and the physicochemical properties of both the flavor and the encapsulating material [173,177]. Table 5 summarizes some examples of effectiveness and beverage applications.

It is noteworthy that excessive starch can destabilize emulsions; optimal ratios are necessary for stability and controlled release. The effectiveness of encapsulation depends on the specific flavor’s properties (volatility, polarity, and molecular size) and the starch derivative used [173,174,176,178]. Moreover, accurate assessment of flavor retention and release requires advanced analytical and modeling techniques [177].

## 10. Haze Prevention in Beers and Other Alcoholic Beverages

Haze formation is a common issue that negatively affects the visual quality and consumer perception of beers and other alcoholic beverages. It results from colloidal instability, driven by macromolecular interactions that cause aggregation and precipitation of proteins, polyphenols, carbohydrates, and microbes. These aggregates scatter light, thereby reducing product clarity (or limpidity) [179]. Among haze-forming compounds, carbohydrates, particularly starch, α-glucans, β-glucans, and arabinoxylans, have been identified as major contributors to colloidal instability in beer [180,181].

Incomplete degradation of starch and non-starch polysaccharides during brewing plays a central role in haze development. Barley starch, composed of amylose and amylopectin linked by α-(1→4) and α-(1→6) glycosidic bonds, constitutes a significant fraction of the beer extract when fully hydrolyzed. However, insufficient starch breakdown, resulting from inadequate malting, improper milling, suboptimal mashing temperature regimes, excessive lautering temperatures, or the carryover of intact starch granules into wort during boiling, can lead to the persistence of starch-derived particles in beer, which contribute to turbidity. Additionally, yeast stress during fermentation may trigger glycogen release, a highly branched glucose polymer structurally similar to amylopectin, thereby further exacerbating haze formation [182].

The insufficient degradation of arabinoxylans is also associated with low extract yield, increased wort viscosity, reduced filtration rates, and haze formation [183]. Similarly, a high β-glucan content in barley may lead to inadequate cell wall degradation during malting, limiting enzyme diffusion, germination, and the mobilization of kernel reserves, ultimately reducing malt extract yield. Residual β-glucan may increase wort viscosity, leading to filtration difficulties during brewing, and may persist into beer maturation, where they contribute to chill haze formation [184]. β-Glucans, which are structural polysaccharides composed of β-(1→3) and β-(1→6)-linked glucose units, can traverse the entire brewing process, from malting to fermentation, and remain in the finished beer [185].

Chill haze is a reversible colloidal turbidity that develops when beer is cooled and is widely regarded as a precursor to permanent haze. It results primarily from interactions among protein degradation products, condensed polyphenols, carbohydrates, and trace minerals, which play modulatory roles. Factors such as low temperatures, oxidative conditions, agitation, light exposure, and the presence of metal ions, such as copper and iron, promote its formation [186,187]. The contribution of arabinoxylans and glucans to haze stability depends strongly on polymer size: low-molecular-weight polymers may stabilize haze particles through hydrogen bonding and increased solubility, whereas high-molecular-weight polymers promote aggregation and precipitation, leading to visible turbidity [188].

Beer haze is considered a major quality defect because clarity is strongly associated with freshness, stability, and overall product quality. Consumers often reject turbid beers, even when haze poses no microbiological risk. Haze can be classified as either visible haze, which is detectable by the naked eye, or submicron (“invisible”) haze, consisting of particles smaller than 0.1 µm. Submicron haze is particularly problematic, as it often precedes visible haze during storage or transportation [189].

The stability of beer is strongly influenced by malt quality, the primary raw material used in brewing. During mashing, inadequate carbohydrate degradation results in the persistence of long-chain dextrins that are not fermentable by yeast. After fermentation, these residual dextrins exhibit limited solubility in alcoholic beverages and contribute to haze formation [190]. Submicron haze particles may originate from altered regions of the starchy endosperm, retrograded starch, and polysaccharides associated with yeast cell surfaces [189]. Carbohydrate-induced haze is particularly difficult to control, as it is not always effectively removed by standard filtration or clarification methods.

Starch and its derivatives play a dual role in beer quality: while they can contribute to haze formation, they may also be exploited to improve colloidal stability depending on their source, structure, and interactions with other beer components [191]. Notably, wort turbidity has been reported to be higher in barley-brewed worts (average 4.9 °EBC, darker worts) than in malt worts (2.4 °EBC, lighter-colored worts). However, the overall turbidity ranges are similar between brewing systems.

Native starches, primarily derived from incomplete hydrolysis of the cereal endosperm (e.g., barley) during malting or mashing, can contribute to haze formation by forming insoluble complexes with other macromolecules (proteins and polyphenols) [160]. Additionally, retrograded starch, re-crystallized amylose, or amylopectin formed during the cooling of gelatinized starch can generate persistent colloidal particles responsible for invisible haze (on the submicron scale). These starch fractions are often not entirely removed during lautering or filtration, especially under suboptimal processing conditions.

Polysaccharides such as residual starch (including α-glucans like amylose and amylopectin), β-glucans, and arabinoxylans (pentosans) from malt, along with minor quantities of yeast-derived glycogen (also an α-glucan), can compromise the colloidal stability of beer and contribute to visible turbidity [191,192,193,194]. Excessive levels of these polysaccharides typically result from poor malt quality, inadequate enzymatic activity, improper milling, or insufficient starch and cell wall degradation during mashing and lautering. Moreover, the presence of glycogen in beer is typically associated with suboptimal yeast management [195].

Among haze-inducing compounds, barley β-glucans are particularly problematic because they form gelatinous aggregates that can clog filters during clarification. Even in the absence of visible gels, β-glucans can significantly contribute to submicron haze [185]. Furthermore, β-glucans and arabinoxylans can interact with polyphenols and proteins through hydrogen bonding, forming complex colloidal aggregates that are difficult to remove and that further exacerbate haze formation [196].

## 11. Prebiotic Alcoholic Drinks

The concept of prebiotics was first introduced by Gibson and Roberfroid [197], who defined them as “nondigestible food constituents that beneficially affect the host by selectively stimulating the growth and/or activity of one or a limited number of bacterial species already resident in the colon” [197]. This definition highlighted the selective nature of prebiotics and their role in modulating gut microbiota. Later, Gibson et al. [198] refined this concept, underscoring the importance of selective fermentation and its impact on microbial composition and activity in the gastrointestinal tract. In 2010, the International Scientific Association for Prebiotics and Probiotics further expanded the definition to include the functionality of prebiotics, describing them as “a selectively fermented ingredient that results in specific changes in the composition and/or activity of the gastrointestinal microbiota, thus conferring benefit(s) upon host health” [199].

Prebiotics are non-digestible food components that support host health by promoting the growth and/or activity of beneficial gut bacteria [200]. They naturally occur in a wide range of plant-based food sources [201]. Among the most well-established prebiotics are fructooligosaccharides, inulin, and galactose- or xylose-containing oligosaccharides. Other fibers with demonstrated prebiotic activity include β-glucans, isomaltooligosaccharides, arabinooligosaccharides, and guar gum [200]. Chitooligosaccharides are derived from chitin, while xylooligosaccharides are sourced from agricultural by-products like corn cobs, rice hulls, malt cakes, and bran [201]. Soybean oligosaccharides are present in soybeans, and β-glucan is predominantly found in oats and barley. Additionally, pectin and plant gums enhance the prebiotic content of many plant foods. Resistant dextrin is typically extracted from corn and wheat, whereas resistant starch occurs naturally in a variety of plant-based foods. Soluble corn fiber, another prebiotic, is explicitly derived from corn. Beyond serving as substrates for microbial fermentation, prebiotics also offer additional health benefits, including anti-inflammatory effects, alleviation of symptoms of intestinal bowel disorders, and potential prevention of colorectal cancer [202].

In recent years, starch and starch derivatives have gained recognition as emerging prebiotic compounds. Resistant starch is a form of starch that is not fully digested in the small intestine and plays a vital role as a prebiotic food. Their structural characteristics, such as the degree of branching and crystallinity, affect the types and amounts of short-chain fatty acids (SCFAs) produced, particularly butyrate and propionate. By serving as a key substrate for colonic fermentation, resistant starch contributes to the production of these advantageous SCFAs [151,152,153,155,203,204].

Although not initially classified as a traditional prebiotic, resistant starch has been proposed as a prebiotic due to its capacity to stimulate the growth of beneficial gut microbiota such as *Bifidobacterium* and *Faecalibacterium*, while inhibiting pathogenic species [152,153,154].

**Figure 4:**
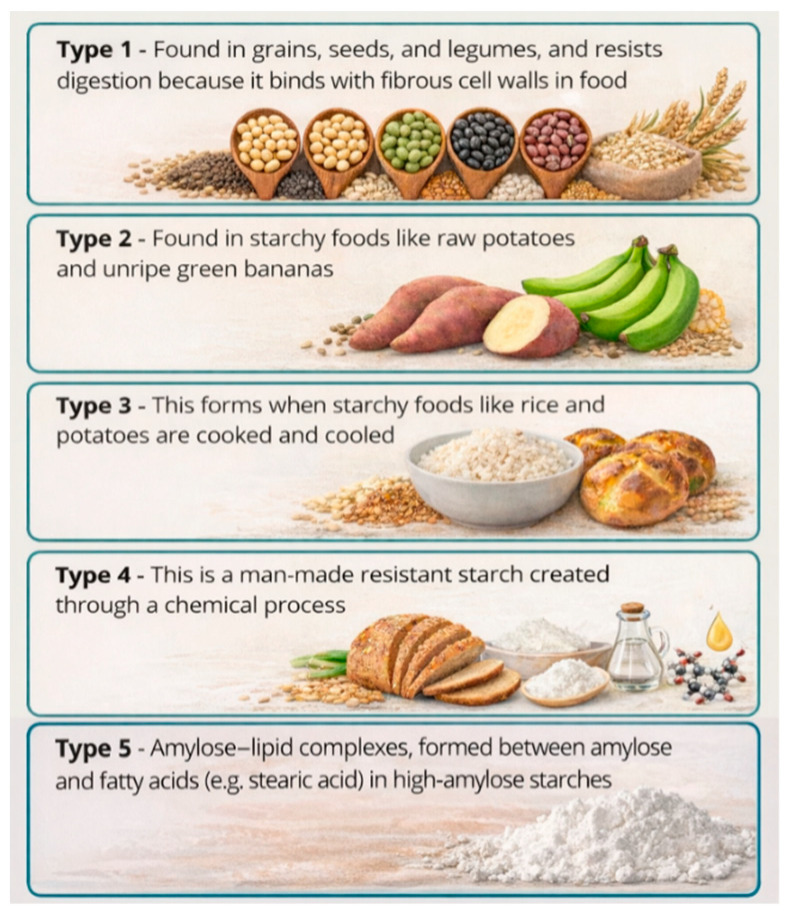
Classification of resistant starch based on its source and structure, and examples of foods where it can be found. Adapted from Raigond et al. [207].

Resistant starch occurs in both whole and processed starch-rich foods. Its content varies significantly across food types; for instance, cereal grains typically contain 7.2–25.2 g/100 g dry matter [205,206]. Food processing methods, such as heating and cooling, can also significantly affect a product’s resistant starch content. Resistant starch is classified into five types based on its source and structure (Figure 4).

In addition to resistant starch, other starch derivatives, such as maltodextrin and resistant dextrin, have also shown prebiotic potential. These compounds resist enzymatic digestion in the upper gastrointestinal tract and serve as fermentable carbon sources for gut microbiota in the colon. Natural food sources rich in these starch-derived prebiotics include whole grains such as wheat, oats, barley, and rye, as well as chicory [208,209]. The prebiotic content in such foods generally ranges from 0.3% to 6% of fresh weight.

The integration of resistant starch and starch-derived fibers into food products underscores their importance in promoting digestive health. Their ability to foster beneficial microbial populations, stimulate the production of health-promoting SCFAs like butyrate, and resist digestion in the upper gastrointestinal tract positions them as key ingredients in functional foods and dietary strategies [205,210,211,212,213].

In the alcoholic beverages industry, starch has traditionally served as a primary carbohydrate source for fermentation, used in the production of beer, spirits, and traditional beverages such as sake [214]. More recently, studies have demonstrated the significant impact of cereal-based beverages on the human gut microbiota [215]. Traditional drinks such as sorghum beer, boza, and burukutu—produced from cereals such as maize, barley, or millet—naturally contain indigestible carbohydrates like β-glucans, arabinoxylans, and resistant starch, all of which have demonstrated prebiotic effects [216]. These fermented cereal-based beverages are widely consumed worldwide and reflect regional grains and fermentation techniques (Table 6). For instance, Boza is a traditional drink made from whole grains, such as maize or flour, and is popular in Turkey, Albania, and Bulgaria [217,218]. Takju, also known as makgeolli, is a cloudy Korean alcoholic beverage made by fermenting rice with nuruk, a starter containing lactic acid bacteria and fungi [219].

Fermented beverages made from cereals such as oats, wheat, maize, rye, millet, sorghum, barley, and rice are increasingly recognized for their health-promoting properties [226,227,228,229]. These grains contain fermentable dietary fibers—such as water-soluble and insoluble arabinoxylans, β-glucans, oligosaccharides, and resistant starch—that serve as substrates for probiotic lactic acid bacteria. When used in fermentation, they deliver the synergistic benefits of both probiotics and prebiotic activity [230].

Cereal-based beverages naturally contain prebiotics due to their inherent indigestible fiber content. For example, fermented oat drinks are rich in β-glucans, which have been shown to reduce LDL cholesterol [231]. Similarly, barley and malt are commonly used as substrates for producing functional beverages [232].

Prebiotic alcoholic beverages represent a novel category of functional drinks that combine traditional consumption patterns with health benefits, particularly by modulating the gut microbiota. These beverages aim to promote the growth of beneficial microbes, such as Bifidobacteria and Lactobacilli, while maintaining a desirable taste and cultural authenticity [200,229]. The inclusion of resistant starch or its hydrolysates provides dual functionality—serving as both a fermentable carbohydrate during production and a prebiotic upon consumption [201,202].

## 12. Final Remarks

Starch and its derivatives, particularly resistant and structurally modified forms, serve as versatile substrates for fermentation processes to produce bioethanol, hydrogen, and other beneficial metabolites. The efficiency and outcomes of fermentation are influenced by the type of substrate utilized, its structural properties, and the optimization of the fermentation process. These factors present significant opportunities for sustainable bioprocessing and health-related applications.

In the alcoholic beverage industry, starch derivatives play a pivotal role as fermentation substrates, stabilizers, clarifying agents, and quality-control tools. Recent advancements in starch modification and enzyme technology have improved production efficiency, enhanced beverage stability, and fostered product innovation, while also addressing critical concerns regarding safety and quality.

The molecular structure of starch—particularly the ratio of amylose to amylopectin, the length of the chains, and the degree of branching—has a substantial impact on fermentation outcomes in alcoholic beverages. Optimizing starch structure can enhance sugar availability, improve fermentation efficiency, increase alcohol yield, and positively influence the sensory attributes of the final product.

Additionally, starch and its derivatives offer practical solutions for flavor stabilization and controlled release in beverages. Their performance depends on the type of starch, the extent of modification, and the composition of the surrounding matrix. While these derivatives provide notable benefits in terms of flavor protection and modulation, precise formulation and a thorough understanding of matrix interactions are imperative for achieving optimal results.

Starch films are transforming beverage packaging by providing biodegradable, functional, and intelligent solutions. They enable spoilage detection, provide edible, dissolvable packaging for instant drinks, and support sustainability efforts in the beverage industry.

Innovation in starch technology is advancing rapidly, yielding significant progress across traditional applications and emerging fields such as 3D food printing. In this context, the unique viscosity and structural stability of starch-based materials are being leveraged to produce customized, intricate food structures that are not only visually appealing but also functionally robust. This intersection of clean-label trends and cutting-edge food technology is catalyzing transformation across the food and alcoholic beverage sectors, enabling the creation of more personalized, natural, and technologically sophisticated products that cater to the evolving expectations of contemporary consumers.

The utilization of starch and its derivatives reflects a broader trend towards nutraceutical and functional beverages. These innovations are a direct response to the increasing consumer demand for health-enhancing, gut-friendly, and culturally relevant beverage options, thereby providing a promising direction for future research and product development.

## Figures and Tables

**Table 1:** Types of starch-based fat replacers used in low-calorie foods and adapted from [42].

Type	Description	Application Example
Native starches	Limited functionality as fat mimics	Basic thickening (soups, sauces)
Modified starches	Cross-linked or substituted for stability	Low-fat dressings, baked goods
Maltodextrins	Short-chain polysaccharides from starch	Creamy texture in dairy or desserts
Resistant starch	Non-digestible, acts as fiber	Calorie reducer, improves texture
Starch-based hydrogels	Water-retaining 3D network	High moisture/fat retention in spreads
Starch-lipid complexes	Emulsified or gelatinous fat mimics	Meat analogues, sauces

**Table 2:** Examples of starch as a coating agent used in the food industry.

Starch’s Type/Polymer Matrix	Additives	Applications	Beneficial Effects	Refs.
Tef starch	Agar (0.4 g), and glycerol (0.30%, *w*/*v*)	Red Crimson Grapes	The packaging and food industry	[7,10]
Corn starch (3.0 or 5.0%, *w*/*w*) and gelatin (10%, *w*/*v*)	Sorbitol or glycerol	Grape	Weight loss and sensory evaluation	[71]
Corn starch (2%, *w*/*v*) and arabic gum (2%, *w*/*v*)	Sorbitol and glycerol	Green banana	Weight loss, firmness, color	[72]
Corn starch	Corncob cellulose and cassia seed oil	Green grapes	Prevented the grapes’ decomposition	[39]
Corn starch	Lecithin and oleic acid	Sunflower oil	Prevented sunflower oil oxidation even after 53 days of storage at 30 °C	[73]
Corn starch	Glycerol and Aloe vera	Tomato	Fruit appearance and weight loss	[74]
Mango kernel starch	Glycerol and Sorbitol	Tomato	Weight loss, firmness, total soluble solids, total titratable acidity, ascorbic acid, fruit decay, and sensory evaluation.	[75]
Cassava starch	Lactic acid bacteria, sodium carboxymethyl cellulose, and glycerin	Bananas	Prevented bananas from browning and turning black.	[76]
Cassava starch	Cinnamon essential oil	Guava	Weight loss, firmness, total and soluble pectin, and pectin methylesterase	[77]
Cassava starch	Pumpkin extract residue and oregano essential oil	Ground beef	Improved antibacterial activity against *E. coli*, *Listeria monocytogenes*, and *Staphylococcus aureus*	[78]
Cassava starch	Potassium sorbate	Strawberry	Firmness, color, sensory evaluation, coating integrity, and respiration rate	[79]
Cassava starch	Glycerol	Fresh-cut mango	Weight loss, respiration rate, firmness, β-carotene content, color, sensory evaluation, and microbiological assays	[80]
Cassava starch (2.0%, *w*/*v*) and chitosan (0.5, 1.0, 1.5, 2.0%, *w*/*v*)	Mixture of *Lippia gracilis* Schauer genotypes and glycerol	Guava	Firmness, color, pH, titratable acidity, total soluble solids	[81]
Rice starch	Glycerin and oregano essential oil	Fish fillets	Improved the microbiological growth in 6 days of storage	[82]
Rice starch	Glycerol, coconut oil, and tea leaf extract	Tomato	Weight loss, total soluble solids, titratable acidity, ascorbic acid content, color, and microbial count	[83]
Rice starch/Fish protein	Glycerol and sorbitol	Strawberry	Coating’s water vapor permeability (sliced carrots), weight loss, firmness, anthocyanin content, surface color, reducing and total sugar content, titratable acidity, soluble, insoluble, and total solids, and microbiological assays.	[84]
Rice starch/Fish protein	Pink pepper phenolic compounds and glycerol	Fresh-cut apples	Color, browning index, firmness, mass loss, total soluble solids, pH, and acidity	[85]
Pea starch (2.5%, *w*/*v*) and guar gum (0.3%, *w*/*v*)	Glycerol, shellac, Tween-20 (0.3 mL), and oleic acid	Orange	Weight loss, firmness, respiration rate, ethylene production, color, acetaldehyde and ethanol concentrations (fruit juice), peel pitting index, fruit decay, stem-end rind breakdown, overall visual acceptability, and sensory evaluation.	[86]
Pea starch (4%, *w*/*v*), potato starch (4%, *w*/*v*), guar gum (1%, *w*/*v*)	Glycerol and Potassium Sorbate	Apple, tomato, and cucumber	Coating weight and thickness, KS residual surface concentration, yeast and mold count	[87]
Pinhão starch	Feijoa peel flour, citric acid, pectin	Apples	Maintained a constant weight after five days of storage	[88]
Purple potato starch	Chitosan	Apples	Maintained the quality of apples for four weeks	[89]
Starch (2%, *w*/*v*)	Citric acid solution (50%, *w*/*v*)	Strawberry	Weight loss, firmness, total soluble solids, color, and total microbial count	[90]
Acetylated tapioca starch	Hydroxyethyl cellulose	Guava	Prolonged the shelf life	[91]
Tapioca starch	Chitosan nanoparticles	Cherry tomatoes	Inhibits the growth of microorganisms	[92]
Thermoplastic starch	Chitosan tripolyphosphate submicron particles containing rosin	Baked goods	The biocomposites were active against *Penicillium roqueforti*.	[93]

**Table 3:** Application area, benefits, and some examples of starch films used in the beverage industry.

Application Area	Description & Benefits	Example/Details	Refs.
Spoilage Detection	Starch films with pH or electrically responsive properties can detect beverage spoilage in real time.	Starch/CMC films change conductivity in response to spoilage in orange juice.	[65,98]
Edible, Water-Soluble Films	Edible packaging film utilizing a combination of κ-carrageenan, carboxymethyl starch, and gum ghatti.	Coffee packaging bags dissolve in 40 s, enhancing the stability of instant coffee.	[99]
Active/Intelligent Packaging	Films can be engineered to have antioxidant, antimicrobial, or spoilage-indicating properties.	The addition of plant extracts or nanoparticles can enhance shelf life and indicate product degradation.	[65,69,100,101,102]
Sustainability	Starch films are biodegradable, renewable, and reduce reliance on petrochemical plastics.	Used for various food and beverage packaging, supporting environmental goals.	[65,99,102,103,104,105]

**Table 4:** Applications & advantages of clean-label starch thickeners.

Application Area	Key Advantages	Refs.
Soups & Sauces	Provide smooth texture, enhance stability, improve mouthfeel, and ensure product consistency even after heat processing and storage.	[140,141]
Dairy Alternatives	Impart creaminess and body, improve stability, and support clean-label claims for plant-based products.	[140,142,143]
Fruit Preparations	Maintain desired consistency, prevent syneresis (water separation), and ensure an appealing appearance throughout shelf life.	[144,145,146]
Bakery Fillings	Enhance structure, retain moisture, and improve sensory quality in fillings and creams.	[131]
Breads, cookies, noodles, and cakes	Improves their physicochemical and textural properties, making products more attractive to consumers—improvements in volume, texture, elasticity, and structural stability.	[146]
Ready-to-Eat Foods & Beverages	Enable instant thickening of cold liquids, provide convenience, and maintain product quality without artificial additives.	[142,147]
3D Food Printing	Offer unique rheological properties for customized, structurally sound, and visually appealing food products.	[115,120,148]

**Table 5:** Key benefits and findings of starch modifications in alcoholic beverages.

Starch Type/Modification	Key Benefits in Beverages	Notable Findings and References
OSA-modified starch (high DS)	High flavor retention, slow release	Retention up to 90%, improved stability [171]
Starch-tannic acid complex	Enhanced binding and retention of off-flavors	Higher affinity for aldehydes [173]
Modified food starch (CAPSUL^®^)	High encapsulation efficiency, stable aroma in hot drinks	92–95% efficiency, aroma stability [174,176]
Native/alkaline-treated starch	Physical entrapment, controlled release	Effective for various volatiles [175]

**Table 6:** Fermented cereal-based beverages. Adapted from: Blandino et al. [220]; Ankita and Bhosale [221]; Kumari et al. [222]; Adesulu-Dahunsi et al. [223]; Vasudha and Mishra [224], and Embashu et al. [225].

Cereal Source	Product	Description	Origin
Maize (Corn)	Busaa	Alcoholic beverage	Nigeria, Ghana
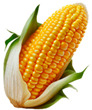	Chikokivana	Alcoholic beverage	Zimbabwe
	Kachasu	Alcoholic beverage	Zimbabwe
	Seketeh	Alcoholic beverage	Nigeria
	Sorghum beer	Acidic, weakly alcoholic liquid drink	South Africa
	Tesgüino	Alcoholic beverage	Northern & North-Western Mexico
	Pito	Alcoholic dark brown drink	Nigeria, Ghana
	Boza	Thick, sweet, slightly sour beverage	Albania, Turkey, Bulgaria, Romania
	Ogi	Fermented beverage	West Africa, Uganda, Kenya, Tanzania
	Kunu	Fermented beverage	Nigeria, Niger
	Chibuku (Shake Shake)	Fermented beverage	Zimbabwe, Zambia, Botswana, Namibia, South Africa
	Chikoko	Fermented beverage	Republic of the Congo
	Goyon	Fermented beverage	São Tomé and Príncipe
	Munkoyo	Fermented beverage	Democratic Republic of the Congo; Zambia
	Kiamu	Fermented beverage	Malawi
	Munkoyo	Fermented beverage	Zambia
	Uji	Fermented beverage	Kenya, Tanzania
	Bilk	Fermented beverage	Namibia
Millet	Bagni	Liquid drink	Caucasus
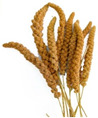	Braga	Liquid drink	Romania
	Busa	Liquid drink	Syria, Egypt, Turkmenistan
	Chikokivana	Alcoholic beverage	Zimbabwe
	Darassum	Liquid drink	Mongolia
	Mangisi	Sweet-sour non-alcoholic drink	Zimbabwe
	Merissa	Alcoholic drink	Sudan; Tanzania
	Togwa	Fermented beverage	East Africa
	Pito	Fermented beverage	Ghana, Nigeria, Togo, Benin
	Ch’titha	Fermented beverage	Algeria
	Bitter Kunu	Fermented beverage	Cameroon
	Kunu	Fermented beverage	Niger, Chad; Kenya, Uganda
	Pombé	Fermented beverage	Angola
	Tchoukoutou	Fermented beverage	Cameroon, Central African Republic
	Atayef	Fermented beverage	Somalia
	Bushera	Fermented beverage	Uganda, Kenya
	Mursik	Fermented beverage	Kenya
	Obushera	Fermented beverage	Uganda
	Rwandan Ikivuguto	Fermented beverage	Rwanda
	Amadumbe	Fermented beverage	South Africa
	Mabele	Fermented beverage	Botswana
Wheat 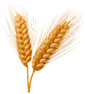	Bouza	Thick, acidic, yellow, alcoholic beverage	Egypt
	Takju	Alcoholic turbid drink	Korea
	Mahewu	Fermented beverage	Africa
	Kishk	Fermented beverage	Egypt
Rice	Brembali	Dark brown alcoholic drink	Indonesia
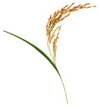	Chongju	Alcoholic clear drink	Korea
	Makgeolli	Turbid rice wine	Korea
	Khaomak	Alcoholic sweet beverage	Thailand
	Sake	Alcoholic clear drink	Japan
	Tapai pulut	Alcoholic dense drink	Malaysia
	Tapuy	Sour-sweet alcoholic drink	Philippines
	Shaosinghjiu	Alcoholic clear beverage	China
	Oshikundu	Fermented beverage	Namibia
	Busa	Fermented beverage	Egypt
Sorghum 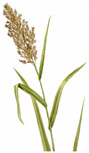	Burukutu	Alcoholic beverage with a vinegar-like flavor	Nigeria, Benin, Ghana
	Otika	Alcoholic beverage	Nigeria
	Merissa	Alcoholic drink	Sudan
	Talla	Alcoholic drink	Ethiopia
	Enjara	Fermented beverage	Ethiopia
	Pito	Fermented beverage	Nigeria, Ghana
	Bissap	Fermented beverage	Sénégal, Guinée, Côte d’Ivoire
	Dolo	Fermented beverage	Burkina Faso, Côte d’Ivoire, Mali
	Gowe	Fermented beverage	Benin, Togo
	Tchakpalo	Fermented beverage	Benin, Togo, Gabon, Equatorial Guinea
	Maaloula	Fermented beverage	Sudan
	T’ej	Fermented beverage	Sudan
	Borde	Fermented beverage	Ethiopian
	Zumbani	Fermented beverage	Zimbabwe
	Sorghum Beer	Fermented beverage	South Africa, Lesotho

## Data Availability

No new data were created or analyzed in this study. Data sharing is not applicable.

## Abbreviations

The following abbreviations are used in this manuscript:

CS	Corn starch
DES	Deep eutectic solvents
DI-FTICR MS	Direct-infusion fourier transform ion cyclotron resonance mass spectrometry
EGCG	Epigallocatechin gallate
EU	European Union
EBC	EBC colour scale, developed by the Institute of Brewing and the European Brewing Convention. Method for colour grading of beers, malts, and caramel solutions, as well as similarly coloured liquids. It has a range of 2 to 27 visual units, yellower pale worts and lagers at the low end of the scale, and the redder colour of dark worts, beers and caramels at the upper end of the scale
FT-IR	Fourier transform infrared spectroscopy
HBE	Honey bee
HPMC	Hydroxypropyl methylcellulose
NADES	Natural-deep eutectic solvents
OS	Octenyl succinylated
OSA	Octenyl succinic anhydride
OSCS	Octenyl succinic anhydride-modified corn starch
O-MTSs	Octenyl succinic anhydride-modified turmeric starches
PR-106	A medium-duration, high-yield rice variety developed by Punjab Agricultural University. It is known for its good milling and cooking qualities and is frequently studied for its physicochemical characteristics.
PR-114	A comparatively early-maturing rice variety, also developed by Punjab Agricultural University, is often evaluated for grain quality, starch composition, and functional properties.
PUSA-44	A high-yielding, long-duration indica rice variety developed by the Indian Agricultural Research Institute (IARI), New Delhi. It is widely cultivated in northern India and commonly used in agronomic and starch-property studies.
RS	Resistant starch
SCFAs	Short-chain fatty acids
SEM	Scanning electron microscopy
TPS	Thermoplastic starch
UPLC-ToF-MS	Ultra-performance liquid chromatography–time-of-flight mass spectrometry
XG	Xanthan gum
XRD	X-ray diffraction
